# Efficacy and tolerability of a specific blend of amino acids in patients with anorexia nervosa treated in a hospital setting: study protocol for a randomized controlled trial

**DOI:** 10.1186/s13063-023-07120-7

**Published:** 2023-02-09

**Authors:** Riccardo Dalle Grave, Simona Calugi, Chiara Ruocco, Mirko Chimini, Agnese Segala, Maurizio Ragni, Michele Carruba, Alessandra Valerio, Enzo Nisoli

**Affiliations:** 1 Department of Eating and Weight Disorders, Villa Garda Hospital, Via Monte Baldo, 89, Garda, 37016 Verona, Italy; 2 Center for Study and Research on Obesity, Department of Biomedical Technology and Translational Medicine, University of Milan, Via Vanvitelli, 32, 20129 Milan, Italy; 3 Department of Molecular and Translational Medicine, Brescia University, Viale Europa 11, 25123 Brescia, Italy

**Keywords:** Anorexia nervosa, Amino acids, Dietary supplement, Lean body mass, Outcome, Physical fitness, Psychopathology, Treatment

## Abstract

**Background:**

Specific blends of essential amino acids (EAA) containing a high percentage of branched-chain amino acids preserves mitochondrial metabolism and higher physical resistance in elderly mice, increasing their survival and improving physical performance and cognitive functions in malnourished elderly patients. However, no study has been yet done on patients with anorexia nervosa (AN) who regain weight with specialized intensive treatment. The present study aims to evaluate the efficacy of supplementation with EAA on the change in lean body mass (LBM) and other physical and psychological outcomes in patients with AN who are undergoing specialist treatment for eating disorders.

**Methods:**

This is a 13-week randomized, double-blind, placebo-controlled study. Patients will be randomized to either a mixture of a complex blend of EAA and intermediates of the tricarboxylic acid (TCA) cycle (citrate, malate, succinate) supplementation (or placebo) upon admission at the intensive residential and day-hospital treatment for eating disorders. Ninety-two participants with AN aged 16–50 years will be recruited from a specialized intensive treatment of eating disorders. Double-blind assessment will be conducted at baseline (T0) and the end of the 13 weeks of treatment (T1). The study’s primary aim is to evaluate the efficacy of supplementation with EAA and TCA intermediates on the change in lean body mass (LBM) with weight restoration in patients with AN who are undergoing specialist treatment for eating disorders. The secondary aims of the study are to assess the effect of dietary supplementation on physical fitness, weight restoration, modification of AN and general psychopathology, and psychosocial impairment.

**Discussion:**

The study’s results will inform researchers and clinicians on whether supplementing a mixture of EAA and TCA cycle intermediates will improve the increase of LBM and other important physical and psychological outcomes in patients with AN who regain weight with specialized intensive treatment.

**Trial registration:**

NCT, NCT05290285. Registered on 22 March 2022.

## Administrative information

**Table:** 

Title {1}	Efficacy and tolerability of a specific blend of amino acids in patients with anorexia nervosa treated in a hospital setting. Study protocol for a randomized controlled trial
Trial registration {2a and 2b}	ClinicalTrials.gov Identifier: NCT05290285
Protocol version {3}	08/1/1/2022, V1
Funding {4}	Professional Dietetics S.p.A (Milan, Italy) will provide the treatment product used in this trial and help cover the costs of some of the analyses
Author details {5a}	^1^Department of Eating and Weight Disorders, Villa Garda Hospital, Via Monte Baldo, 89, 37016 Garda (Verona), Italy ^2^Center for Study and Research on Obesity, Department of Biomedical Technology and Translational Medicine (BIOMETRA), University of Milan, Via Vanvitelli, 22, 20129, Milan, Italy ^3^Department of Molecular and Translational Medicine (DMMT), Viale Europa, 11, Brescia University, Brescia, Italy
Name and contact information for the trial sponsor {5b}	Riccardo Dalle Grave (rdalleg@gmail.com) is the Principal Investigator and contact person of the trial sponsor (Villa Garda Hospital)
Role of sponsor {5c}	The present study is investigator-initiated. Professional Dietetics S.p.A had no role in study design and will not be involved in data collection, analysis, interpretation, or publication. The authors will not receive financial or non-financial support for the research or article authorship

## Introduction

### Background and rationale {6a}

Anorexia nervosa (AN) is a severe eating disorder (ED) characterized by restriction of energy intake relative to requirement, leading to a significantly low weight, overvaluation of shape and weight, and persistent lack of recognition of the seriousness of the current low body weight. It has the highest mortality rate among all psychiatric diseases [1]. Multidisciplinary treatment incorporating medical, psychological, and nutritional interventions is the best practice for these patients [2, 3]. However, the underlying pathophysiology is unclear, and the outcome of recommended treatments is often unsatisfactory [4]. A recent systematic meta-review of meta-analyses and network meta-analyses has confirmed that no medication effectively reduces ED and general psychopathology and improves weight outcome in patients with AN [5]. These data support previous evidence that drugs cannot be recommended to improve body weight or specific psychopathology [4, 6] unless psychiatric comorbidity is present. On the other hand, weight restoration and the recovery of a normal nutritional status are key effective elements in the treatment of AN [7, 8], similarly to muscle and cognitive function improvement [9].

Assessment of nutritional aspects is crucial in the treatment of AN because evidence has shown that even beyond weight restoration, patients continue to show nutritional insufficiencies [10], a more limited spectrum of food preferences [11], and restricted dietary intake [12]. A recent systematic review and assessment of evidence quality on dietetic interventions for adult outpatients with an ED concluded that across the studies examining participants with AN, there was poor quality of evidence for the majority of outcomes measured (i.e., weight, ED behaviors, and psychopathology). They concluded that it is currently problematic for ED clinicians to draw firm conclusions about what should be enclosed in dietetic treatment for outpatients with AN [13].

On the other hand, nutrition is one of the significant determinants of health, particularly in AN. Among the three primary macronutrients, proteins—in particular, their quantity and quality (i.e., their specific amino acid profile)—play a crucial role in regulating metabolic health and longevity. Amino acids (AAs) are essential not only for protein synthesis and influencing nutritional status but also as sources of vital elements (e.g., nucleotides, neurotransmitters) and as signal molecules for the modulation of gene expression and epigenetic mechanisms [14].

Chronic food supplementation with a specific blend of essential amino acids (EAA) containing a high percentage of branched-chain amino acids (leucine, isoleucine, valine) preserves mitochondrial metabolism and greater physical resistance in elderly mice, increasing their survival [15]. Amino acid mixtures can also counteract the functional atrophy of skeletal muscles in elderly and cachectic or sarcopenic individuals [16, 17].

We have recently demonstrated the greater efficacy of new EAA blends enriched with intermediates of the Krebs cycle on energy metabolism [18]. In particular, we found a beneficial effect on physical and cognitive performance in SAMP8 mice supplemented with a specific EAA formula enriched with Krebs cycle intermediates (PD-E07) [19]. This mouse model of functional decline related to the age of skeletal muscle and impaired working memory is widely used to evaluate pharmacological, nutritional, or exercise efficacy. It has been shown that the PD-E07 formula, administered orally with drinking water (1.5 mg/kg) in addition to the regular diet, improves muscular and cognitive performance after 3 months of treatment, promoting mitochondrial rejuvenation in the striated muscles and hippocampus.

More specifically in humans, a recent randomized study was conducted for 2 months, in a double-blind, on 155 malnourished older adults (aged > 80 years), in which a group of patients (*N* = 78) received a dietary supplement with a specific formula of EAA (8 g / day) plus nutritional advice and one group (*N* = 77) received placebo plus nutritional advice. The study found that the EAA supplement increased physical performance and improved cognitive functions in malnourished elderly patients by promoting the bioenergetics of mitochondria in circulating peripheral blood mononuclear cells (PBMCs) [20].

To our knowledge, only one study has been conducted with AA on patients with AN. The study, a double-blind, randomized controlled trial, had a duration of 3 weeks and evaluated the effect of the semi-essential amino acid L-tyrosine (100 mg/kg/day) versus a placebo supplement on cognition and emotional state in 19 female patients with severe and long-lasting AN treated in a hospital setting [21]. Tyrosine shortened reaction time and test duration in memory tasks and improved depressive mood. No side effects have been reported with the use of tyrosine. To date, no studies have evaluated the effect of supplementation with AAs in patients with AN managed with a treatment aimed to address weight restoration and the remission of the eating disorder psychopathology.

We hypothesize that patients with AN receiving the AminoTher-Pro™ product (corresponding to PD-E07; hereafter, AmT-P) compared to those who receive a placebo will achieve the following at the end of the treatment: (i) a higher percentage increase in LBM; (ii) a more significant improvement in physical fitness; (iii) a more significant reduction in ED psychopathology, general psychopathology, and psychosocial injury secondary to the ED; and (iv) a more significant change in the bioenergetic functions in PBMCs, circulating metabolites, and metagenomic profile of the fecal microbiome.

### Objectives {7}

The study aims to compare the efficacy of dietary supplementation with the designer product (AmT-P)—compounded by an original EAA mixture and TCA cycle intermediates with precise stoichiometric ratios—to placebo on LBM modifications obtained with weight restoration in AN patients undergoing a specialist ED treatment.

The secondary aims of the study are to assess the efficacy of the dietary supplement in improving physical fitness, weight regains, modification of ED and general psychopathology, and psychosocial impairment, compared to placebo supplementation.

### Trial design {8}

This study is a randomized, double-blind, placebo-controlled superiority trial. Patients will be randomized to either AmT-P or placebo supplementation upon admission to the treatment. Randomization is carried out at the hospital entry by adopting a stratification for the following parameters: (i) age (16–50); and (ii) body mass index (BMI) (BMI > 14 kg/m^2^). The treatment lasts 13 weeks.

## Methods: participants, interventions, and outcomes

### Study setting {9}

The study will be performed at the Department of Eating and Weight Disorder of the Villa Garda Hospital (Garda, Italy), a community clinic accredited with the Italian National Health System for the inpatient and day-hospital rehabilitation treatment of eating disorders.

### Eligibility criteria {10}

Participants meeting the following criteria will be included in the trial: diagnosis of AN, age between 16 and 50 years, and written informed consent.

Exclusion criteria are as follows: schizophrenia or other psychotic disorders, bulimia nervosa, substance use disorder, medical complications that are potentially capable of hindering the interpretation of the results (e.g., a medical disease that induces weight loss), presence of physical treatments (including drugs) that are potentially able to hinder the interpretation of the results (e.g., chemotherapy for cancer), and pregnancy or intention to become pregnant during treatment.

The study will be performed by medical doctors, psychotherapists, and dietitians.

### Who will take informed consent? {26a}

The physicians of the ED unit will obtain informed consent or assent from potential trial participants or authorized surrogates. Informed consent will be given verbally and in writing, dated and signed by the subject, or in the case of a minor patient, by their parents and/or legal guardians.

### Additional consent provisions for collection and use of participant data and biological specimens {26b}

Not applicable. We have described the terms of collection and use of participant data and biospecimens in the informed consent.

## Interventions

### Explanation for the choice of comparators {6b}

Participants will be randomized to the experimental formula AmT-P or placebo. An inert placebo is the correct comparator as the research serves to establish the safety and the efficacy is unknown. Future trials may include active comparators if collected data suggest clinical benefit. Placebo is an isocaloric product containing maltodextrins rather than AAs [22].

### Intervention description {11a}

The composition of the AmT-P mixture (AminoTher-Pro™, Professional Dietetics S.p.A., Milan, Italy, Italian food supplements register code 95,457) is shown in Table 1. The AmT-P blend will be administered orally at 4.5 g twice daily (two sachets per day).

**Table 1:** Composition of the amino acid mixture (PD-E07/AminoThe-Pro^TM^)

	PD-E07/AminoTher-PRO^TM^		
	1 sachet (g)	2 sachets (g)	g/100
l-Leucine	1.2	2.4	23.5
l-Lysine (chlorhydrate)	0.9	1.8	17.6
l-Isoleucine	0.6	1.2	11.7
l-Valine	0.6	1.2	11.7
l-Threonine	0.7	1.4	13.7
l-Cysteine	0.2	0.3	2.9
l-Histidine	0.2	0.3	2.9
l-Phenylalanine	0.1	0.2	2–0
l-Methionine	0.1	0.1	1.0
l-Tryptophan	0–1	0.1	1.0
Vitamin B1 (thiamine chlorhydrate)	0.0007	0.0001	0.0
Vitamin B6 (pyridoxine chlorhydrate)	0.0009	0.0002	0.0
Citric acid	0.409	0.82	8.0
Malic acid			1.92
Succinic acid	0.103	0.21	2.0
Total EAA (g)	4.5	9.0	100.0
Total mixture (g)	5.1	10.2	

Participants will take two sachets per day, orally, under the supervision of the nurse at mid-morning and midafternoon the day after the randomization. Boxes of the experimental sachets will be stored in a locked cabinet before administration in climate-controlled conditions and monitored weekly for consistency in ambient temperature.

The proposed formula consists of a complex blend of essential AAs, including some precursors of neurotransmitters that are important for regulating mood. In particular, phenylalanine, a precursor of tyrosine (non-essential AA), is involved in the biosynthesis of catecholamines (noradrenaline, adrenaline, dopamine), and tryptophan is the precursor of serotonin. Furthermore, the AAs of the mixture and the intermediates of the TCA cycle (citrate, malate, succinate) may stimulate mitochondrial bioenergetics by improving metabolism and muscle function.

#### Intensive treatment of eating disorders

The intensive treatment for EDs applied at the Department of Eating and Weight Disorders of Villa Garda Hospital has been described in previous publications [23–25]. The intervention is based on the enhanced cognitive behavioral therapy of eating disorders (CBT-E) [26], a treatment recommended by the National Institute for Health and Care Excellence guidelines for all eating disorders in adults and adolescents [2].

The treatment lasts 13 weeks and can be delivered in an inpatient or a day-hospital setting. The only difference between the two treatments is that in the day hospital, patients sleep outside the unit. Both treatments last 13 weeks and are delivered 7 days a week. The program is personalized to address the eating disorder psychopathology and its maintaining mechanisms operating in the patient. A distinctive characteristic of intensive CBT-E is its collaborative nature and the non-use of “coercive” or “prescriptive” attitudes or procedures. The goal is to engage patients in treatment and change throughout treatment actively. For this reason, admission to intensive CBT-E is collaboratively decided with the patients after three to four preparatory sessions. Since patients are expected to address weight restoration from the first day of the treatment, they must agree on this goal before they are admitted.

The main procedures of intensive CBT-E are shown in Table 2. These are delivered by a “non-eclectic” multidisciplinary team (physicians, psychologists, nurses, and dieticians) trained in the CBT-E. Clinical psychologists provide the procedures for addressing ED psychopathology in individual sessions, which occur twice a week in the first 4 weeks, once a week afterward, and twice in psychoeducational groups twice a week. A dietitian assists patients during meals until they achieve a BMI of ≥ 18.5. Physicians manage the patient’s physical health, and nurses supervise the administration of medications, assist patients in collaborative weighing, and help them to manage significant crises and daily difficulties. The last 3 weeks of the treatment are devoted to concluding the treatment well and preparing for the transition to outpatient therapy.

**Table 2:** Core procedures and nutritional and physical rehabilitation protocols of intensive CBT-E

Core procedures
• Assisted eating (three meals and one snack a day)
• Weekly review meeting (with the clinical psychologist, dietician, nurse, and physician)
• Collaborative weighing one a week
• Individual CBT-E sessions
• Group treatment sessions
Nutritional rehabilitation protocol
• Weight goal: BMI ≥ 19
• Expected speed of weight gain: 1 to 1.5 kg per week
• Daily calorie content of the meal plan is established collaboratively with the patient according to the following guidelines:
—Week 1: Menu A (1500 kcal)
—Week 2: Menu B (2000 kcal)
—Afterward:
○ If the weight increases between 1 and 1.5 kg per week, the meal plan is maintained with the same calorie content as the previous week
○ If the weight increases to less than 1 kg per week, the meal plan is increased by 500 kcal per day. For example, from Menu B to Menu C (2500 kcal) or from Menu C to Menu D (3000 kcal)
○ If the weight increases more than 1.5 kg per week, the meal plan is reduced by 250 kcal per day. For example, from Menu C to Menu B / C (2250 kcal)
○ When the patient reaches a BMI of 19, the program’s goal is to identify a weight range of 3 kg that can be achieved without adopting a calorie restriction. In most patients, weight maintenance occurs with a diet between 2000 (Menu B) and 2500 kcal (Menu C)
Physical rehabilitation protocol
• All patients above a BMI of 15 participate in two 30-min sessions per week of calisthenics with a physiotherapist

*Abbreviations*: *BMI* Body mass index, *CBT-E* Enhanced cognitive behavioral therapy

### Criteria for discontinuing or modifying allocated interventions {11b}

Patients may withdraw from the trial at any time for any reason. The AmT-P mixture was demonstrated as safe and tolerable at the same dosage and length in malnourished older adults [20]. We do not anticipate that severe harm to require early product discontinuation or medical intervention. However, participants will be withdrawn by the team involved in the trial if they experience any new-onset, moderate, or severe adverse events from the intervention. The trial will be terminated if > 20% of all participants exhibit adverse events of moderate or great severity which are new from baseline or progressively worsening.

No intervention dose modifications or administration frequency changes are allowed. Participants will be considered non-adherent and withdrawn from the trial if deviations from the dosing standard are reported to the study team.

### Strategies to improve adherence to interventions {11c}

Adherence will be monitored daily throughout the trial, as the sachets of the placebo and the EAA supplementation will be administered by the team nurse and taken by the participants in front of them. Participants will be considered adherent by taking > 80% of expected doses throughout their participation in the trial. The study product will be stored in a locked cabinet in a locked room for future quality assessment.

### Relevant concomitant care permitted or prohibited during the trial {11d}

Participants will be allowed antidepressants, benzodiazepines, mood stabilizers, and atypical antipsychotics to manage psychiatric comorbidly and multivitamins during the treatment. Any protein and AA supplements and anabolic drugs are prohibited during the trial.

### Provisions for post-trial care {30}

There is no anticipated harm and compensation for trial participation.

### Outcomes {12}

#### Primary outcome

Change of LBM with weight regain in patients with AN. The assessment of body composition will be performed at baseline (T0), at the end of the 13 weeks of treatment (T1), and at 24 weeks follow-up (T2) with the dual-energy X-ray absorptiometry (DXA) (Prodigy Primo Lunar, A223040501, General Electric Company, Madison. WI 53,707–7550, USA-EnCORE TM 2009 (v13.31) software). The DXA has been used in previous studies assessing the changes of LBM in patients with AN [27]. The Bioelectrical Impedance (BIA) (Tanita HealthWare® Native Software, Tokyo, Japan) will be used simultaneously to improve body composition measurement accuracy. Indeed, we will measure total body proteins with the method suggested by [28], which associates the DXA with the BIA data, which has good accuracy in evaluating total body water in patients with AN [29].

This primary outcome will be of extreme clinical importance as LBM deficit and abnormalities beyond total body skeletal muscle mass are associated with several severe medical repercussions in other populations, such as low bone mineral density [30], reduction in physical fitness (i.e., strength, metabolic function, and athletic performance) [31], more extended hospitalization [32], and high rates of mortality [33].

#### Secondary outcomes

The secondary outcomes of the study include the changes between T0 and T1 of the following physical and psychological features:

- Physical fitness measured with the Eurofit Physical Fitness Test Battery (EPFTB) [34].
- Physical activity measured with the Actiheart system, CamNtech Ltd., Cambridge, UK
- Weight regain measured with the Seca Digital Wheelchair Scale Model 664 (Seca, Hamburg, Germany).
- ED psychopathology assessed with the Eating Examination (EDE) Interview [35, 36], the EDE Questionnaire (EDE-Q) [37, 38], and the Eating Problem Check List (EPCL) [39].
- General psychopathology assessed with the Brief Symptom Inventory (BSI) [40, 41].
- Clinical impairment secondary to EDs assessed with the Clinical Impairment Assessment (CIA) [42, 43] and the Starvation Symptom Inventory (SSI) [44].

The measurement of the above physical features was chosen as secondary outcomes because they represent potential mediators of the efficacy of the EEA mixture in improving the LBM. At the same time, improving the eating disorder and general psychopathology and reducing the clinical impairment is the primary goal of treating AN.

Other outcomes of the study include the changes between T0 and T1 of the following:

- Plasma metabolome and miRNAome [45].
- Mitochondrial mass and bioenergetics parameters (mtDNA, enzymatic functions of respiratory chain complexes), transcriptome (RNA-seq), methylome.
- Fecal metagenome. [46, 47].
- Urine metabolome.

The exams were chosen as secondary outcomes to improve our understanding of the underlying mechanisms of action of the AmT-P mixture in changing the LBM.

### Participant timeline {13}

**Fig. 1:**
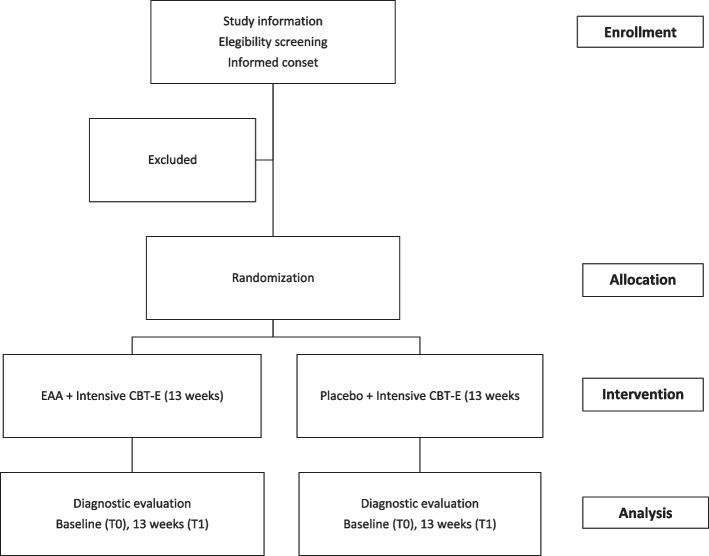
Illustration of the participant flow through the study. CBT-E = enhanced cognitive behavior therapy

Patients will be screened with an in-person clinical screening visit by a study physician on the day of admission to the unit (T0) to determine initial eligibility. If the candidate is eligible, they are evaluated on the same day in randomization visits. All additional visits of the physician will occur biweekly for the duration of the intervention and follow the procedure flow according to Fig. 1 and Table 3.

**Table 3:**
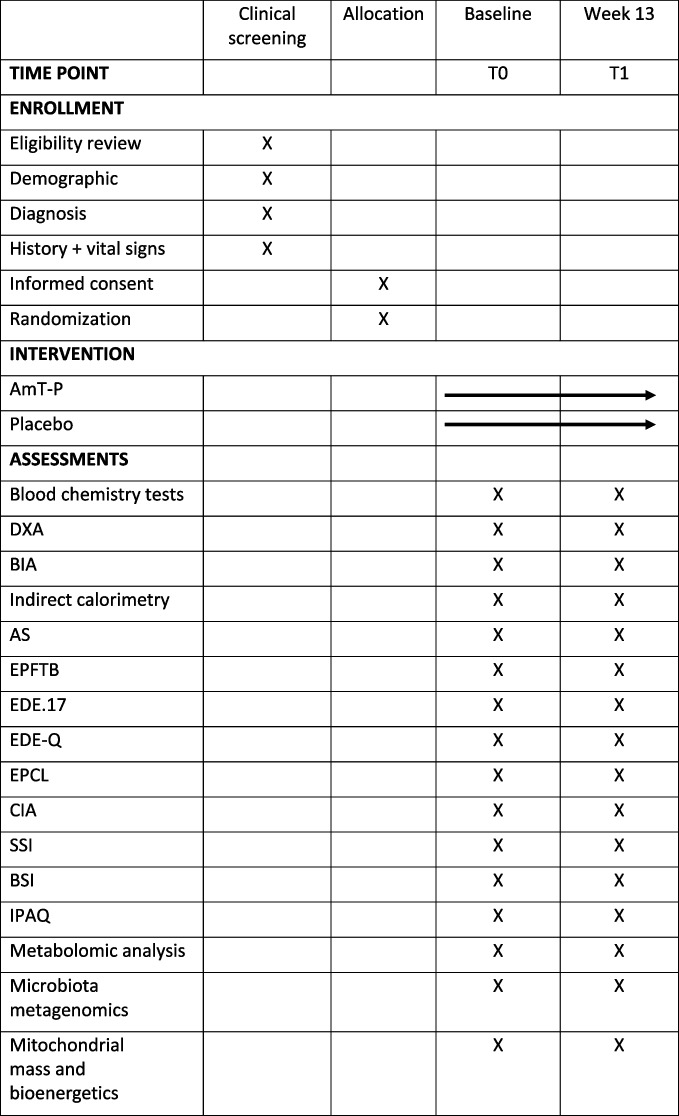
Study schedule of enrolment, interventions, and assessments

*DXA* Dual-energy X-ray absorptiometry, *BIA* Bioelectrical impedance analysis, *AS* Actiheart System, *EPFTB* Eurofit Physical Fitness Test Battery, *EDE.17* Eating Disorder Examination Interview, *EDE-Q* Eating Disorder Questionnaire, *EPCL* Eating Problem Check List, *CIA* Clinical Impairment Assessment, *SSI* Starvation Symptom Inventory, *BSI* Brief Symptom Inventory, *IPAQ* International Physical Activity Questionnaire

### Sample size {14}

Since this is an efficacy study, the primary data evaluation will be the analysis of completers. The primary outcome of interest is the percentage increase in the patient’s LBM with weight recovery at the end of the intensive treatment. The sample size was determined based on the difference between the intervention group and the control group on the change in LBM between baseline and end of treatment. We hypothesized, referring to the data of our previous study [27], that the control group could have an 8.8% increase in LBM, while the intervention group could have a further 2% increase in LBM [48, 49] to reach a variation of LBM of 10.8%. The standard deviation assumed for the sample size calculation is 3.2%.

Based on these premises, will be necessary to have a group of completers of 80 patients at the end of the treatment (i.e., 40 patients per treatment condition). To calculate the initial sample needed to generate 80 patients at the end of treatment, we considered the intervention interruption rate (set at 15%). This calculation results from an initial sample of 92 patients (46 per treatment condition).

### Recruitment {15}

The Department of Eating and Weight Disorder of Villa Garda Hospital accepts about 70 adult patients with AN per year. All the patients are referrals from their family doctors.

Study participants will be recruited among patients who meet the diagnostic criteria for AN, assessed with the EDE.17 interview [35, 36] consecutively admitted at the inpatient or day-hospital rehabilitative of the Department of Eating and Weight Disorder of Villa Garda Hospital.

On the day of admission into the Unit, the patient will meet the ward physician to collect the medical history, the physical examination, and the prescription for humoral and instrumental analyses. On this occasion, the enrollment phase will take place, including the complete data collection for the compilation of the data collection form. At the end of the data collection, if the criteria for inclusion in the study are met, the patient will receive detailed information on the following:

- Study procedures and objectives.
- Purposes, methods, inconveniences, risks, and benefits that the study may entail.
- Freedom to participate and withdraw from the study without affecting subsequent treatment.
- Compensation procedures and treatment of any damages deriving from participation in the study.

Before requesting to sign the informed consent and the possibility of using (and having permission to use) the information collected during the study, the patient will be asked to read an information sheet containing detailed information on the study.

If the patient or, in the case of a minor patient, his parents and/or legal guardians agree to participate in the study, they will have to sign the informed consent form and the authorization to process personal and sensitive data.

Supposing that the patient meets the criteria for inclusion in the study but refuses to participate, she will continue the intensive treatment at the Department of Eating and Weight Disorder of Villa Garda Hospital.

After the interview and signing of the informed consent and data processing, the patient will be randomized to AmT-P or placebo supplementation. Both arms will receive the same intensive treatment for the eating disorder.

At the end of the interview with the patient, a letter will be sent to the family doctor, giving information on the study that the patient will address.

## Assignment of interventions: allocation

### Sequence generation {16a}

The randomization will be performed by the statistician (SC) of the Villa Garda Hospital, Garda (VRI). The randomization sequence will be computer-generated and stratified according to BMI and age into the following four groups:

- Group 1: BMI ≤ 15.2 & age ≤20
- Group 2: BMI > 15.2 & age ≤ 20
- Group 3: BMI ≤15.2 & age > 20
- Group 4: BMI > 15.2 & age > 20

### Concealment mechanism {16b}

Use of a validated password website will ensure concealment.

### Implementation {16c}

The trial sponsor and the principal investigator team will generate randomization with the allocation sequence.

## Assignment of interventions: blinding

### Who will be blinded {17a}

Blinding will be accomplished through multiple methods. Trained staff uninvolved in trial operations or analyses will produce a group code, receive and label product bottles accordingly, and generate stratified randomization sequences using freely available software. Upon randomization, participants will be assigned a new alphanumeric ID reflecting their enrollment status. All parties involved in trial conduct will remain blinded until the primary trial analyses are deemed complete by all trialists.

### Procedure for unblinding if needed {17b}

Unblinding will be permissible only in the case of a severe adverse event requiring hospitalization or resulting in a fatality.

## Data collection and management

### Plans for assessment and collection of outcomes {18a}

#### Data collection form

The examining physician will complete data collection at the time of enrollment. It includes some general information (marital status, level of education, work activity, family and personal medical history for obesity and related diseases, and drug history) and diagnostic criteria for diagnosing AN according to DSM-5 criteria. All data must precisely reflect the patient’s point of view, that is, be an expression of her knowledge about her and her family’s health.

#### Treatment progress evaluation form

It will include the name of the therapists who conduct the treatment, the date of admission and discharge, the body weight and height measured, and any psychopharmacological therapy.

#### Dropout form

It will report the date of dropout from the treatment and the reasons as described by the patient for the voluntary interruption of therapy.

#### Randomization form

It will report the patient’s ID and the two parameters (age and BMI) required for stratification.

#### Blood chemistry tests

They will include the following tests:

- Fasting blood glucose, triglyceridemia, HDL, total VLDL and LDL cholesterol, C reactive protein, fasting insulin, ALT, AST, ALP; GGT, GFR, NA, K, Ca, P, and Vitamin D-25OH.
- Metabolomic analysis (*n* = 15–20 patients/group) [45]. Plasma and urine samples will be collected at T0 and T1, immediately frozen on dry ice, and stored at − 80° C. The samples will then be manipulated for “targeted” metabolomic analysis (glycolysis, TCA cycle, amino acid metabolism) by high-resolution LC–MS liquid chromatography-mass spectrometry (LC–MS), in addition to the analysis of amino acid levels with Biochrom30^+^ (amino acid analyzer).
- miRNome analysis (*n* = 15–20 patients/group) [45]. Plasma and urine samples will be collected at T0 and T1, immediately frozen on dry ice, and stored at − 80 °C. The samples will then be manipulated to analyze miRNome (Next Generation Sequencing, NGS system).
- Mitochondrial mass and bioenergetics (*n* = 15–20 patients/group). Peripheral blood samples will be collected at T0 and T1. The circulating PBMCs will be isolated within 2 h of collection, pelleted, and immediately frozen on dry ice, then stored in biobank. Samples will be manipulated to analyze the following parameters: (i) mtDNA content, (ii) enzymatic functions of the respiratory chain complexes, (iii) production of ATP, (iv) transcriptome (RNA-seq method), and (v) methylome (whole-genome DNA methylation analysis) [20].
- Metagenomic analysis of the microbiota (n = 15-20 participants) [46, 47]. Stool samples will be collected at T0 and T1, immediately frozen in dry ice, and stored at − 80 °C. The samples will then be manipulated for metagenomics analysis.

#### Medical devices

- Dual-energy X-ray absorptiometry (DXA) (Prodigy Primo Lunar, A223040501, General Electric Company, Madison, WI 53,707–7550, USA-EnCORE TM 2009 (v13.31) software) will be used to measure LBM, and also total and regional fat mass and bone mass. Scans will be performed in the morning, and no special preparations will be made except to instruct participants to wear only underwear and no metal accessories.
- Bioelectrical Impedance (BIA) (Tanita HealthWare® Native Software, Tokyo, Japan) will be used to evaluate total body water on the same day the DXA scans are obtained. Participants are asked to take off their clothes, wear a hospital gown, and remain upright before stepping on the tool. They will be then instructed to stand on the scale barefoot in a wide, comfortable position with their arms relaxed and to the side, looking forward and remaining as relaxed as possible. Body composition data will be estimated using software provided by the manufacturer of the bioelectrical impedance analyzer.
- Seca Digital Wheelchair Scale Model 664 (Seca, Hamburg, Germany) will measure the weight and the height from which the BMI will be calculated.
- Vmax Encore 229 Sensormedics system (indirect calorimetry) to measure the Resting Energy Expenditure (REE). After a 12-h fast, participants lie supine in a quiet, dark room for 30 min before measuring VO_2_ and VCO_2_. The last 15 min of which will be also used to determine REE.
- *Actiheart system*, CamNtech Ltd., Cambridge, UK. It is a heart rate monitor combined with a uniaxial accelerometer. The monitor is applied to the left side of the chest and worn day and night for four consecutive days. Participants will be instructed to continue their usual activities, and the time spent in moderate to intense physical activity (≥ 3 MET) will be analyzed.

#### Physical fitness tests

- Eurofit Physical Fitness Test Battery (EPFTB) [34]. The EPFTB is a set of fitness tests that include (i) aerobic fitness (6-min walking test); (ii) musculoskeletal fitness (standing broad jump test, handgrip test, sit-up test); (iii) flexibility (sit-and-reach flexibility test); and (iv) motor fitness (flamingo balance test).

#### Eating disorder and general psychopathology

These components will be assessed with the following measures:

- EDE.17 interview [35, 36]. It is an interview validated in Italian that evaluates the severity of the ED psychopathology of the eating disorder in the last 28 days and allows you to make an accurate diagnosis of anorexia nervosa and other eating disorders according to the diagnostic criteria of the DSM-5.
- EDE Questionnaire [37, 38]. It is a self-reported questionnaire validated in Italian that assesses the severity of eating disorder psychopathology in the last 28 days.
- Eating Problem Check List (EPCL) [39]. It is a self-reported questionnaire validated in Italian that assesses ED attitudes and behaviors in the last 7 days.
- Clinical Impairment Assessment (CIA) [42, 43]. It is a self-reported questionnaire validated in Italian that assesses the severity of clinical impairment secondary to the eating disorder psychopathology.
- Starvation Symptom Inventory (SSI) [44]. It is a self-reported questionnaire validated in Italian that assesses the main symptoms secondary to starvation in the last 28 days.
- Brief Symptom Inventory (BSI) [40, 41]. It is a self-reported questionnaire validated in Italian that assesses the severity of psychological distress in the last 7 days.

### Plans to promote participant retention and complete follow-up {18b}

A maximum effort will be made to engage participants in as many assessments as possible and achieve a high adherence rate in the study. Appointments for the follow-up assessments will be offered at times that best suit the participants. Patients will be provided breaks during examinations, and the assessments can be split into several shorter sessions if required. The research team will electronically monitor participants’ adherence to the assessment schedules, using the treatment progress evaluation form. Retention rates will be continuously monitored by the principal investigator (RDG) during the entire course of the study.

### Data management {19}

#### Data forms and data entry

In the study, all data will be entered electronically. This will be done at the participating site where the data originated. Original study forms will be entered and kept on file at the participating site. Participant files will be stored in numerical order and in a secure and accessible place and manner. Participant files will be maintained in storage for a period of 5 years after the completion of the study.

#### Data transmission and editing

The data entry screens will resemble the paper forms approved by the Ethical Committee. Data integrity will be enforced through a variety of mechanisms. The option to choose a value from a list of valid codes and a description of what each code means will be available where applicable. Checks will be applied at the time of data entry into a specific field and/or before the data is written (committed) to the database. Modifications to data written to the database will be documented through either the data change system. Data entered into the database will be retrievable for viewing through the data entry applications. The type of activity an individual user may undertake will be regulated by the privileges associated with their user identification code and password.

#### Data discrepancy inquiries and reports to core coordinating centers

Additional errors will be detected by programs designed to detect missing data or specific errors in the data. These errors will be summarized along with detailed descriptions for each specific problem in Data Query Reports, which will be sent to the Data Manager.

The Data Manager who will receive the inquiry will respond by checking the original forms for inconsistency, checking other sources to determine the correction, and modifying the original (paper) form entering a response to the query. Note that it will be necessary for Data Managers to respond to each inquiry received to obtain closure on the queried item.

The Data Manager and participating site personnel will be responsible for making appropriate corrections to the original paper forms whenever any data item is changed. Written documentation of changes will be available via electronic logs.

#### Security and back-up of data

All forms related to study data will be kept in locked cabinets. Access to the study data will be restricted. A password system will be utilized to control access. These passwords will be changed regularly.

A complete back-up of the database will be performed twice a month. These tapes will be stored off-site in a climate-controlled facility and retained indefinitely. Incremental data back-ups will be performed on a daily basis. These tapes will be retained for at least 1 week on-site. Back-ups of periodic data analysis files will also be kept. These tapes will be retained at the site. In addition to the system back-ups, additional measures will be taken to back up and export the database on a regular basis at the database management level.

#### Description of hardware

The primary access to the department computing facility will be through the Internet. For maximum programming efficiency, the SPSS statistical analysis systems will be employed for this study. Security is enforced through passwords and may be assigned to groups and individuals at different levels.

### Confidentiality {27}

All study-related information will be stored securely at the study site. All participant information will be kept in locked file cabinets in areas with limited access. All laboratory specimens, reports, data collection, process, and administrative forms will be identified only by a coded ID [] number to maintain participant confidentiality. All records that contain names or other personal identifiers, such as locator forms and informed consent forms, will be stored separately from study records identified by code number. All local databases will be secured with password-protected access systems. Forms, lists, and any other listings that link participant ID numbers to other identifying information will be stored in a separate, locked file in an area with limited access. All blood chemistry, physical fitness, and psychological test results will be kept strictly confidential, and all counseling and blood draws will be conducted in private rooms.

Participants’ study information will not be released outside of the study without the participant’s or legal guardian's written permission (if necessary).

### Plans for collection, laboratory evaluation, and storage of biological specimens for genetic or molecular analysis in this trial/future use {33}

The collection, processing, and storage of biological specimens (blood, urine, feces) will be performed according to established international standard operating procedures as outlined by the national and international node of the European Biobank and BioMolecular Resources Research Infrastructure (BBMRI.it, BBMRI-ERIC) and respecting the rights of the patients and citizens involved.

Each patient will be identified with a unique alphanumeric code. This code will be associated with all the aliquots of biological materials, with a unique identification code per aliquot.

Biological samples will be collected at the Villa Garda Hospital at T0 and T1, immediately frozen in dry ice, and sent in dry ice at the collaborating centers for subsequent analyses. Plasma, urine, and fecal samples will be sent at BIOMETRA (University of Milan) and kept at − 80 °C until analyses or for long-term storage. The PBMC aliquots will be sent to DMMT (University of Brescia) and stored in the Department’s cryogenic bank until analyses or for long-term storage.

#### Blood samples

All recruited subjects will be given a blood draw of 20 mL at T0 and T1.

Blood draw will be processed within 2 h after collection and analyzed as follows:

- Peripheral blood mononuclear cell (PBMC) preparation. Blood will be collected in EDTA-coated tubes and then diluted with an equal volume of PBS + 2% FBS (Immunological Science, Bolney, Sussex) and divided into two identical aliquots. Diluted blood will be slowly layered on top of the Lymphoprep™ gradient (V = 1.077 g/ml; Stemcell Technologies, Vancouver, Canada) and centrifuged (400 × *g*, 35 min at room temperature, with the break off). PBMC layer will be collected in a new tube, washed in PBS + 2% FBS (100 × *g* for 10 min, the break off) to remove platelets, and then washed a second time (400 × *g* for 10 min, the break on). The PBMC pellet will be immediately frozen. The mitochondrial functions will be evaluated by measuring ATP production and enzyme functionality of respiratory chain complexes. The mitochondrial mass measurement [i.e., the mtDNA amount], the gene expression profile (i.e., RNA-seq), and the epigenomic characterization (i.e., methylome analysis) will be conducted to elucidate the mitochondrial biology, energy metabolism, and immunological phenotyping.
- Plasma samples will be used to measure the free amino acid profile as a biomarker of metabolic, cardiovascular, immune, and inflammatory diseases. Further, targeted metabolomics, miRNome, and circulating mtDNA will be evaluated.

#### Urine samples

All recruited subjects will be given a urine sample of 10 mL at T0 and T1. Metabolomics and miRNoma analysis will be performed.

#### Fecal samples

All recruited subjects will be given a fecal sample of 300 mg at T0 and T1. The metagenomic analysis will be performed.

## Statistical methods

### Statistical methods for primary and secondary outcomes {20a}

At the end of the treatment, the LBM of the patient will be assessed, the blood chemistry and instrumental tests will be evaluated, and the scores of each questionnaire used will be calculated, assessing the effects of the treatment on the scores of each scale.

The univariate analysis (*t*-test for independent samples) will allow us to evaluate the differences at the end of treatment (T1) in the percentage of LBM between the intervention group and the control group. In addition, with mixed linear models, it will be possible to evaluate the difference between the two treatment arms in the primary and secondary outcomes at the end of therapy. Mixed linear models will be able to incorporate all available data collected in the study and effectively treat missing data commonly present in longitudinal studies. Models will be compared using maximum likelihood estimation rather than limited maximum likelihood estimation, as the focus is on the whole model, including fixed and random effects. The main clinical features (i.e., BMI, ED psychopathology, and general psychopathology) will be included in the models as potential predictors of the trajectories of change.

The analyses will also be controlled for physical activity and its variation between baseline and end of hospitalization (measured with the Actiheart system and IPAQ).

### Interim analyses {21b}

No interim analyses were planned.

### Methods for additional analyses (e.g., subgroup analyses) {20b}

A secondary analysis of the primary endpoint will adjust for those pre-randomization variables which might reasonably be expected to be predictive of favorable outcomes (i.e., baseline LBM scores). Statistical inference will be based on one-sided *P* values and 95% confidence intervals which adjust for the stopping rule used for the primary analysis.

### Methods in analysis to handle protocol non-adherence and any statistical methods to handle missing data {20c}

We will test superiority using two analysis sets; the intention-to-treat set, considering all patients as randomized regardless of whether they received the randomized treatment, and the “per protocol” analysis set. Criteria for determining the “per protocol” group assignment will be established by Data Manager before the trial begins.

#### Imputation procedure for missing data

We will report reasons for withdrawal for each randomization group and compare the reasons qualitatively. Any missing data’s effect on results will be assessed via sensitivity analysis of augmented data sets. Dropouts will be included in the analysis by modern imputation methods for missing data.

The main feature of the approach is creating a set of clinically reasonable imputations for the respective outcome for each dropout. This will be accomplished using a set of repeated imputations created by predictive models based on the majority of participants with complete data. The imputation models will reflect uncertainty in the modeling process and inherent variability in patient outcomes, as reflected in the complete data.

After the imputations are completed, all of the data (complete and imputed) will be combined, and the analysis performed for each imputed-and-completed dataset. Rubin’s multiple (i.e., repeated) imputation method will be used to estimate the treatment effect. We propose to use 15 datasets (an odd number to allow use of one of the datasets to represent the median analytic result).

These methods are preferable to simple mean imputation, or simple “best–worst” or “worst-worst” imputation, because the categorization of patients into clinically meaningful subgroups, and the imputation of their missing data by appropriately different models, accords well with best clinical judgment concerning the likely outcomes of the dropouts, and therefore will enhance the trial’s results.

### Plans to give access to the full protocol, participant-level data, and statistical code {31c}

The datasets analyzed during the current study and statistical code will be available from the corresponding author on reasonable request, as be the full protocol.

## Oversight and monitoring

### Composition of the coordinating center and trial steering committee {5d}

The coordinating center is the Department of Eating and Weight Disorders of Villa Garda Hospital, Garda (VR), Italy. It will be responsible for running the trial day-to-day, including recruitment and data collection of all data, and management. RDG will provide supervision regarding the introduction and closing sessions. Clinical investigators, and study coordinators, will meet weekly.

### Composition of the data monitoring committee, its role and reporting structure {21a}

The data monitoring committee will be independent of the sponsor and will be composed of a medical doctor specialized in endocrinology, a medical doctor specialized in pharmacology, a psychologist, and a biostatistician. The data monitoring committee will review regular reports produced by the study team to ensure safe and responsible conduct of the trial and make any determination of discontinuation.

### Adverse event reporting and harms {22}

Adverse events will be formally assessed and graded according to severity at each biweekly medical visit via spontaneous reports of the participants. Adverse events reported by participants between study medical visits will be termed “spontaneous adverse events.” If an adverse event is of moderate or greater severity, the study team will coordinate with the patient’s physician and develop and document a response or monitoring plan.

The principal investigator (RDG) will evaluate the event’s causality to the trial intervention. Any event assessed as possibly, probably, or definitely related is classified as related to the trial intervention. The principal investigator (RDG) will make a severity assessment of the event as mild (i.e., a tolerable complication), moderate (i.e., it interferes with daily activities), or severe (i.e., it makes daily activities impossible). All serious adverse events will be documented and reported immediately (within a maximum of 24 h) to the principal investigator (RDG) and the health director of the Department of Eating and Weight Disorders of Villa Garda Hospital, Garda (VR). If it cannot be excluded that the severe adverse effect is attributable to the intervention under investigation, the principal investigator (RDG) will report it to the Ethics Committee of Verona and Rovigo, Italy, within 15 days.

### Frequency and plans for auditing trial conduct {23}

The Ethics Committee of the Verona and Rovigo, Italy, can visit the research sites for a trial auditing. On that occasion, it can have direct access to the data and study files. Involved parties will be kept data participants strictly confidential.

### Plans for communicating important protocol amendments to relevant parties (e.g., trial participants, ethical committees) {25}

Substantial amendments are changes that affect participants’ safety, health, rights, and obligations; changes in the protocol that affect the study objective(s) or central research topic; and changes in the study leader and sponsor. Substantial protocol amendments will be submitted to the Ethics Committee of Verona and Rovigo, Italy, for approval before implementation.

### Dissemination plans {31a}

We aim to make the trial findings accessible to researchers, clinicians, people, and their families affected by AN. Therefore, we will disseminate the results through open-access scientific journals, conference presentations, social media, and relevant websites addressing the topic of EDs.

## Discussion

The treatment outcomes of AN are still suboptimal. Indeed, less than 50% of patients achieve complete and lasting remission with the best treatment available in outpatient [50] and intensive settings [51]. These unsatisfactory results indicate the available treatments need to be modified to make them more effective.

The use of EAA blends enriched with intermediates of the Krebs cycle has been found to determine a beneficial effect on physical and cognitive performance in SAMP8 mice [19] and improve physical performance and cognitive functions in the malnourished elderly patient [20]. These data indicate that this nutritional supplementation added to the usual treatment care might improve the outcome of patients with AN. In particular, they might enhance the increase of LBM and other physical and psychological outcomes such as physical fitness, eating disorder, and general psychopathology. However, no data are available on the effect of this supplement mixture on patients with AN.

This study is the first that will examine the efficacy of a mixture of EAAs and intermediates of TCA cycle supplementation in patients with AN treated with an intensive specialized treatment aimed to restore their body weight and improve their eating disorders and general psychopathology.

The data of the study will help both clinicians and researchers. Clinicians will have data on a robust randomized control trial on the clinical utility to supplement with EAAs and patients with AN who are addressing weight regain and their psychopathology with EAAs and TCA cycle intermediates. The multiple outcome measures evaluated in the trial at the end of treatment and 24 weeks follow-up will permit to improve the knowledge on the short- and middle-term effects of this supplementation not only for the improvement of LBM but also for other key clinical outcomes, such as physical fitness, weight regain, and, most of all, eating disorder and general psychopathology and psychosocial impairment associated with AN.

The metabolomic analysis in plasma and urine, the measurements of mitochondrial mass and bioenergetics parameters, and the metagenomic analysis of the microbiota in faces will be helpful for researchers to have more knowledge about the underlying biological mechanisms of the observed clinical changes associated with EAAs and TCA intermediates supplementation in patients with AN during the process of weight regain.

In conclusion, the results of this study will inform clinicians and researchers if the supplementation of a mixture of EAAs and intermediates of the Krebs cycle will be able to improve the increase of LBM and other important physical and psychological outcomes in patients with AN who regain weight with specialized intensive treatment.

### Trial status

The study protocol was approved by the Ethics Committee of Verona e Rovigo, Italy, on 4 March 2022 (Prot. n. 15,087). Recruitment starts start in September 2022, and the expected duration of enrollment is 78 weeks–18 months.

## Data Availability

The datasets used and/or analyzed will be available from the corresponding author upon reasonable request after the study is complete.

## Abbreviations

AA: Amino acid
AmT-P: AminoTher-Pro™
AN: Anorexia nervosa
BIA: Bioelectrical impedance analysis
BMI: Body mass index
BSI: Brief Symptom Inventory
CBT-E: Enhanced cognitive behavioral therapy of eating disorders
CIA: Clinical Impairment Assessment
DXA: Dual-energy X-ray absorptiometry
EEA: Essential amino acid
EDE: Eating Disorder Examination
EDE-Q: Eating Disorder Examination Questionnaire
EPFTB: Eurofit Physical Fitness Test Battery
EPCL: Eating Problem Check List
IPAQ: International Physical Activity Questionnaire
SSI: Starvation Symptom Inventory
PBMC: Circulating peripheral blood mononuclear cell
